# Medical Department of the University of the Pacific

**Published:** 1859-10

**Authors:** 


### MEDICAL DEPARTMENT OF THE UNIVERSITY OF THE PACIFIC.

We are in receipt of the Introductory Address, delivered by Hon. George Barston, at the opening of the Medical Department of the University of the Pacific, in California, with (under the same cover,) addresses by the Rev. Dr. Peck and Rev. M. Cutler. Containing many valuable thoughts, worthy those who gave them utterance, and the great occasion which gave them birth. We hail with joy this uprising temple in the glowing West, as another monument to the progress and stability of our glorious Institutions; and as a welcome beacon, whose light shall dispel the last vestage of ignorance and superstition, so long holding undisputed sway over a land burthened with heaven's richest blessings. And we are pleased to see the right-hand of fellowship, with the most cordial greeting, extended to this *Summer* School of Medicine—particularly as it comes from some who have been loud in their denunciations of such a course of instruction heretofore, as a heinous offence against established usage. Truly "'tis distance lends enchantment," &c., or "*Tempus mutat.*"

